# Characterization of Zeolite in Zeolite-Geopolymer Hybrid Bulk Materials Derived from Kaolinitic Clays

**DOI:** 10.3390/ma6051767

**Published:** 2013-05-06

**Authors:** Hayami Takeda, Shinobu Hashimoto, Hiroaki Yokoyama, Sawao Honda, Yuji Iwamoto

**Affiliations:** Department of Environmental and Materials Engineering, Nagoya Institute of Technology, Gokiso-cho, showa-ku, Nagoya 466-8555, Japan; E-Mails: shinobuh@nitech.ac.jp (Sh.H.); hyw6nj@yahoo.co.jp (H.Y.); honda@nitech.ac.jp (Sa.H.); iwamoto.yuji@nitech.ac.jp (I.Y.)

**Keywords:** geopolymer, zeolite, kaolin, compressive strength

## Abstract

Zeolite-geopolymer hybrid materials have been formed when kaolin was used as a starting material. Their characteristics are of interest because they can have a wide pore size distribution with micro- and meso-pores due to the zeolite and geopolymer, respectively. In this study, Zeolite-geopolymer hybrid bulk materials were fabricated using four kinds of kaolinitic clays (a halloysite and three kinds of kaolinite). The kaolinitic clays were first calcined at 700 °C for 3 h to transform into the amorphous aluminosilicate phases. Alkali-activation treatment of the metakaolin yielded bulk materials with different amounts and types of zeolite and different compressive strength. This study investigated the effects of the initial kaolinitic clays on the amount and types of zeolite in the resultant geopolymers as well as the strength of the bulk materials. The kaolinitic clays and their metakaolin were characterized by XRD analysis, chemical composition, crystallite size, ^29^Si and ^27^Al MAS NMR analysis, and specific surface area measurements. The correlation between the amount of zeolite formed and the compressive strength of the resultant hybrid bulk materials, previously reported by other researchers was not positively observed. In the studied systems, the effects of Si/Al and crystalline size were observed. When the atomic ratio of Si/Al in the starting kaolinitic clays increased, the compressive strength of the hybrid bulk materials increased. The crystallite size of the zeolite in the hybrid bulk materials increased with decreasing compressive strength of the hybrid bulk materials.

## 1. Introduction

When kaolinitic clays are heated at approximately 700 °C, metakaolin with an amorphous phase is obtained. The metakaolin is often activated by mixing with alkali hydroxide solution and water-glass, as a result of which a polycondensation reaction occurs, leading to the formation of cementitious material with high mechanical strength and high durability. This consolidated material is commonly known as a geopolymer [1]. Metakaolins derived from kaolinitic clays with different particle size, purity and crystallinity yield geopolymers with different properties [2]. Thus, it is important to take these factors into account when fabricating geopolymers using metakaolin as a starting material.

Zeolite is sometimes found in geopolymers, especially when the curing temperature is above 85 °C [2,3,4]. The amount of zeolite increases with curing time [4,5]. The fabrication of zeolite-geopolymer hybrid bulk materials has already been achieved, and their characteristics are of particular interest. Since zeolites have high microporosity and geopolymers have mesopores [6], zeolite-geopolymer hybrid bulk materials can have a wide pore size distribution. As such, zeolite-geopolymer hybrid materials with arbitrary properties can be used, for example, as building materials having the excellent property of humidity control.

In this study, zeolite-geopolymer hybrid materials were fabricated using four kinds of kaolintinc clays (a halloysite and three kinds of kaolin). The crystalline phase of kaolintic clays and the ^29^Si and ^27^Al MAS NMR and specific surface area of matakaolin were investigated. The amounts and species of zeolite, compressive strength and pore distribution of the resultant zeolite-geopolymer hybrid bulk were investigated. Furthermore, it was discussed, which property of the kaolin or metakaolin affects the generation of zeolite and the compressive strength of the resultant hybrid materials.

## 2. Experimental

### 2.1. Materials Treatment

Four kinds of kaolinitic clays, *viz*. a halloysite (K0) and three kinds of kaolinite (K1, K2, and K3), were used. The chemical compositions and Si/Al atomic ratios of these kaolinitic clays are tabulated in Table 1. These kaolinitic clays were calcined at 700 °C for 3 h to transform the metakaolin into amorphous aluminosilicate phases [7]. The metakaolins derived from K0, K1, K2, and K3 will hereinafter be denoted by MK0, MK1, MK2, and MK3, respectively. The formulation of zeolite-geopolymer hybrid bulk materials are shown in Table 2. The samples were fabricated from a slurry mixture composed of one of four kinds of metakaolin (MK0, MK1, MK2, and MK3), ground to a mean particle size of about 10 μm, a sodium silicate solution (SiO_2_: 37%; Na_2_O: 17.5%; Kishida Chemical Industries, Ltd., Fukuoka, Japan), sodium hydroxide (99%; Kishida Chemical Industries, Ltd., Fukuoka, Japan) and distilled water. Commercial X-type zeolite (Zeorum F9: TOSO Co. Ltd.,Tokyo, Japan) was also prepared. The bulk bodies derived from MK0, MK1, MK2, MK3, and MK3 with commercial X-type zeolite will be denoted by BK0, BK1, BK2, BK3, and BK3Z, respectively. The starting slurry mixture was cast into a cylindrical acrylic mold (Φ15 mm × 30 mm high) with one side closed and maintained at 80 °C and 50% relative humidity for 72 h inside a temperature-humidity control (THC) chamber.

**Table 1:** Chemical compositions of the kaolinitic clays used (mass%).

Sample	SiO_2_	Al_2_O_3_	Fe_2_O_3_	TiO_2_	CaO	MgO	K_2_O	Na_2_O	Ignition loss	Si/Al (mol)
K0	48.12	36.33	0.33	0.16	0.04	-	0.03	0.05	14.80	1.17
K1	42.66	40.92	1.12	0.45	0.14	0.04	0.09	0.14	14.13	0.92
K2	40.86	39.87	0.39	0.46	0.12	0.12	0.17	0.01	17.91	0.91
K3	49.35	36.03	0.20	0.02	0.02	0.02	2.29	0.04	11.94	1.21

**Table 2:** The starting mixing ratios of the bulk materials (mass%).

Sample	MK0	MK1	MK2	MK3	Commercial X-type zeolite	Sodium silicate solution	NaOH	Distilled water
BK0	100	-	-	-	-	60	27	60
BK1	-	100	-	-	-	60	27	60
BK2	-		100	-	-	60	27	60
BK3	-	-	-	100	-	60	27	60
BK3Z	-	-	-	70	30	60	27	60

### 2.2. Analysis

The micromorphology of the samples was examined using a scanning electron microscope (SEM: JSM-6360LVS; JEOL Co., Ltd., Tokyo, Japan). The crystalline phases of the initial kaolinitic clays and the final bulk products were determined by powder X-ray diffraction analysis (XRD: XD-D1; Shimazu Co., Ltd., Tokyo, Japan). Quantitative analysis of the NaP- or NaX-type zeolite in the final bulk products was performed on the basis of master curves obtained by high-precision XRD analysis using a NaP-type zeolite derived from an NaA-type zeolite (Zeorum A-4: TOSO Co. Ltd.,Tokyp, Japan) [8] and NaX-type zeolite (Zeorum F-9: TOSO Co. Ltd.,Tokyo, Japan). Quantitative analysis was performed using an internal standard method. The crystallite sizes of the zeolites were calculated using Scherrer’s equation. The specific surface area of each metakaolin and final bulk materials measured by the N_2_ adsorption method using a FlowSorb III (Micromeritics Instrument Corp., Norcross, GA, USA). Solid-state ^27^Al and^ 29^Si MAS NMR spectra of metakaolin and final bulk materials were obtained using a UNITY Inova 400 plus (Varian Co., Ltd., Palo Alto, CA, USA) spectrophotometer. ^27^Al and^ 29^Si NMR magic angle spinning (MAS) spectra were obtained by operating at 104.2 MHz and 79.6 MHz, respectively. The chemical shifts were calibrated with respect to the zero reference of AlCl_3_•6H_2_O for ^27^Al and of tetramethylsilane (TMS) for ^29^Si. The compressive strength of the samples was measured using an INSTRON5582 (INSTRON Co., Ltd., Norwood, MA, USA). The crosshead speed used was 10 mm/min. Three to five test pieces were prepared for each condition. The pore volume and pore size distribution of the samples were evaluated using N_2_ sorption isotherm analysis (Model: Autosorb-1, Quantachrom Instrument, Boynton Beach, FL, USA). In particular, the porosity of the bulk samples were characterized by the BJH [9] method. Table 3 shows the analysis conducted for different materials.

**Table 3:** Analysis conducted for different materials.

Sample	SEM	XRD	Specific surface area	Compressive strength	Pore size distribution
Kaolintic clays	■	■	□	□	□
Metakaolin	□	□	■	□	□
Bulk materials	■	■	■	■	■

## 3. Results and Discussion

### 3.1. Characterization of Initial Kaolinitic Clays and Their Metakaolins

**Figure 1:**
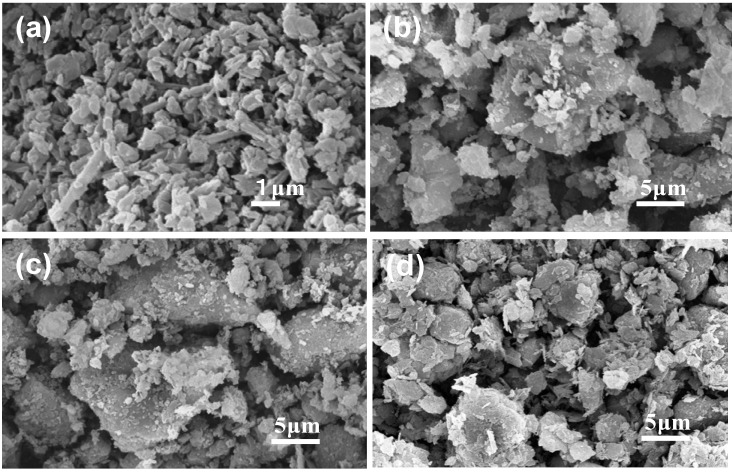
SEM micrographs of the initial kaolinitic clays: (**a**) K0; (**b**) K1; (**c**) K2; and (**d**) K3.

**Figure 2:**
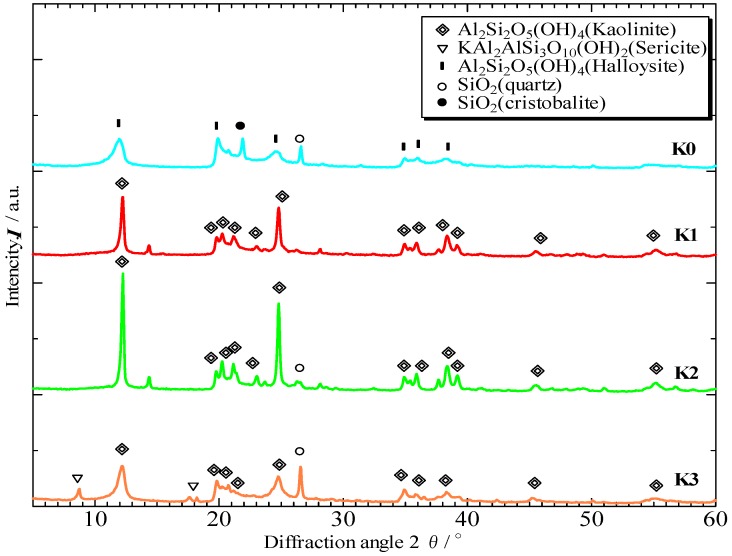
X-ray diffraction patterns of the initial kaolinitic clays K0, K1, K2, and K3.

Figure 1 shows the micromorphology of the initial kaolinitic clays K0, K1, K2, and K3. K0 showed pillar-like particles, whereas K1, K2, and K3 showed plate-like particles. The X-ray diffraction patterns of the initial kaolinitic clays, shown in Figure 2, revealed that halloysite had low crystallinity, and the kaolinitic clays had varying degrees of crystallinity depending on the composition. K2 had a higher-crystallinity kaolinite compared to the other clays. Cristobalite and quartz were observed in K0 as additional crystalline phases, whereas quartz was the only additional crystalline phase in K2. K3 contained a sericite and quartz in addition to kaolinite.

**Figure 3:**
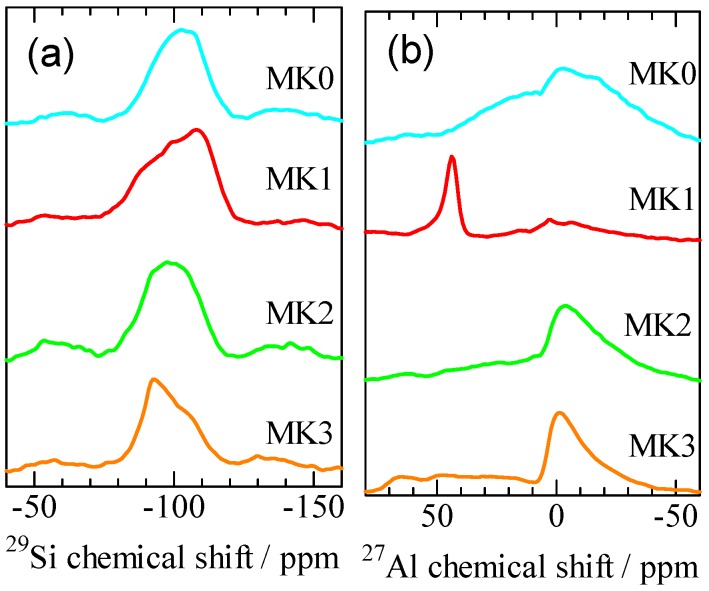
(**a**) ^29^Si; and (**b**) ^27^Al NMR spectra of the metakaolins MK0, MK1, MK2, and MK3.

Figure 3 showed the ^29^Si and ^27^Al NMR spectra. In the ^29^Si NMR spectra of MK0, MK1, MK2, and MK3, approximately –100 ppm peaks were detected for all samples. These peaks, often observed in metakaolins, were assigned to the three-fold coordination: Q^3^ [10]. With increasing number of A1 combine with the peak should shift to higher values* i.e.*, to left in Figure 3 [11]. Thus, Si in MK3 seemed to be combining with more Al atoms than that in MK1. In the ^27^Al NMR spectra, the 0, 25–30, and 50–60 ppm peaks are assigned to six- [Al(VI)], five- [Al(V)], and four-fold coordination [A1(IV)] states, respectively [12,13]. The ^27^Al NMR spectra of MK0 showed a broad peak from –50 to 50 ppm, suggesting that MK0 contained Al(VI), Al(V), and A1(IV) states. The ^27^Al NMR spectra of MK1 exhibited a 50 ppm peak indicating that MK1 contained mostly A1(IV). The ^27^Al NMR spectra of MK2 and MK3 showed a 0 ppm peak attributable to the A1(VI) state. Thus, MK2 and MK3 contained more A1(VI) states than did MK1. The specific surface areas of MK0, MK1, MK2, and MK3 were 23.2, 5.3, 15.6, and 12.5 m^2^∙g^−1^, respectively.

### 3.2. Formation of Zeolite and Geopolymer in Bulk Materials

**Figure 4:**
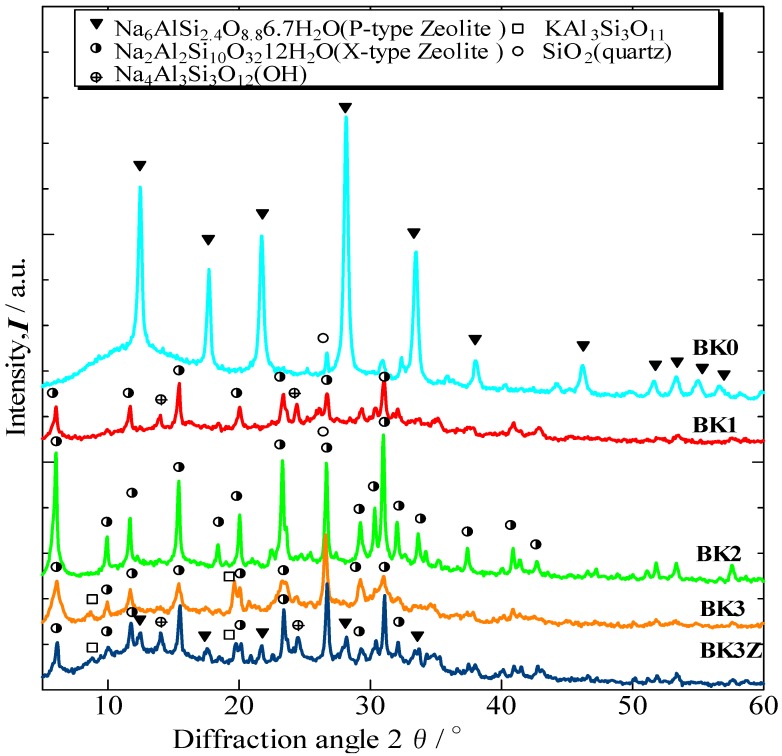
X-ray diffraction patterns of the final bulk materials (BK0, BK1, BK2, BK3, and BK3Z).

Figure 4 shows the X-ray diffraction patterns of the bulk materials obtained after alkali activation treatment (BK0, BK1, BK2, BK3, and BK3Z). X-type zeolite was detected in the kaolinite-derived BK1, BK2, BK3, and BK3Z, showing that X-type zeolite can be produced from kaolinite. In contrast, P-type zeolite was detected in BK0, indicating that P-type zeolite can easily be formed from halloysite. Generation of different type of zeolite could occur from the difference in crystal structure between halloysite and kaolinite.

**Figure 5:**
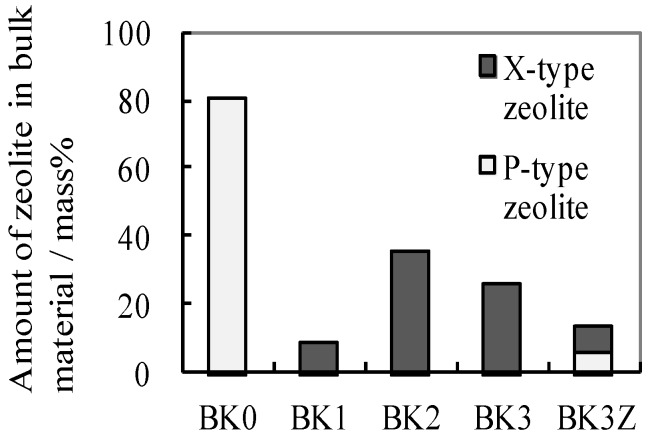
Amount of zeolite in the final bulk products.

As shown in Figure 5, BK0 contained 80.6 mass% Na-P type zeolite. BK1, BK2, BK3, and BK3Z contained 8.3, 35.3, 25.3, and 8.2 mass% Na-X type zeolite, respectively. BK3Z, which was fabricated from metakaolin (MK3) with commercial X–type zeolite, contained a smaller amount of X-type zeolite than did BK3, which was fabricated without addition of the commercial X-type zeolite. BK3Z also contained P-type zeolite and hydroxysodalite. Thus, the commercial X-type zeolite appeared to have transformed into the more stable P-type zeolite and hydroxysodalite [14] during alkali-activation treatment. These results showed that the addition of commercial X-type zeolite into the starting material did not increase the content of X-type in the final bulk material.

**Figure 6:**
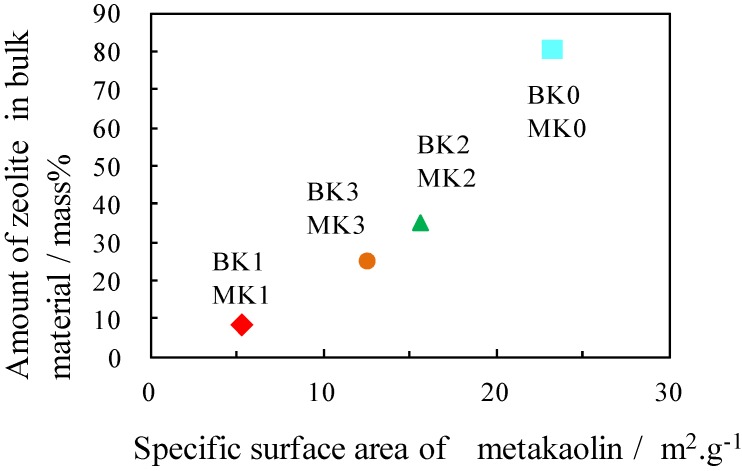
Relationship between the specific surface area of metakaolin and the amount of zeolite in the final bulk materials.

Figure 6 shows the relationship between the specific surface area of metakaolin and the amount of zeolite in the final bulk body. The amount of zeolite increased with increasing specific surface area. The higher the specific surface area was, the higher the solubilities of the Al and Si ions were, resulting in an increase in zeolite formation.

**Figure 7:**
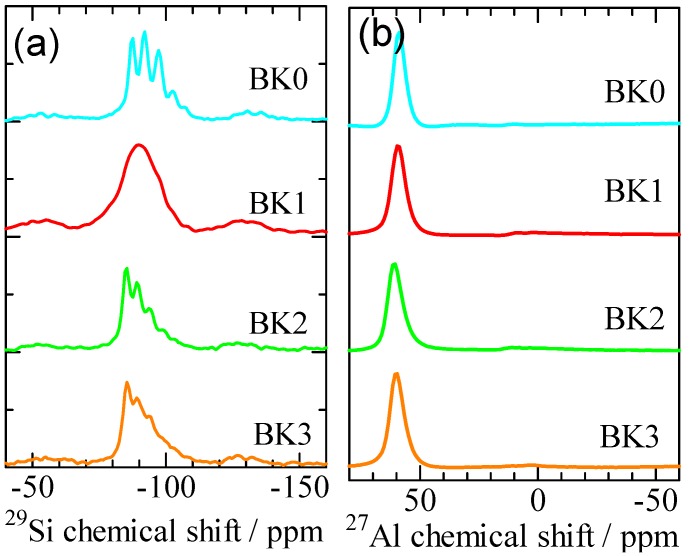
(**a**) ^29^Si; and (**b**) ^27^Al NMR spectra of the final bulk bodies BK0, BK1, BK2, and BK3.

Figure 7 shows the ^29^Si and ^27^Al NMR spectra of the final bulk materials BK0, BK1, BK2, and BK3. In Figure 7a, sharp chemical shifts were observed for ^29^Si NMR except in the case of BK1. The peaks observed at the chemical shifts –87, –92, –97, –102, and –107 ppm in BK0 are attributed to P-type zeolite [9]. The peaks at the chemical shifts –85, –89, –94, –99, and –103 ppm in BK2 and BK3 correspond to X-type zeolite [15]. Since the amount of zeolite in BK1 was low (see Figure 5), no sharp chemical shifts were observed. In the ^27^Al NMR shown in Figure 7b, only a peak approximately 60 ppm which assigned to Al(IV) [13] was identified. As the geopolymerization is usually accompanied by an increase in Al(IV) and a decrease in Al(VI) [16], these results show that the geopolymerisation has been achieved in these samples. The difference of Al-coordination in metakaolin, as shown in Figure 3b, did not show obvious effects on the progress of geopolymerization and the Al-coordination in the products.

### 3.3. Strength and Microstructure of Materials

**Figure 8:**
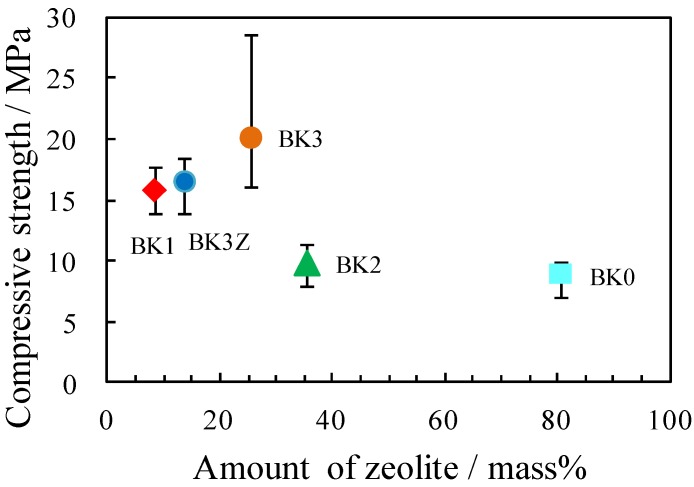
Relationship between the compressive strength and the amount of zeolite in the final bulk materials.

The compressive strength values of BK0, BK1, BK2, BK3, and BK3Z were 8.9, 15.8, 9.9, 20.2, and 16.6 MPa, respectively. Figure 8 shows the relationship between the amount of zeolite and the compressive strength of the bulk materials. It has been reported that increasing the amount of zeolite in the geopolymer reduces strength [3,5]. However, in the present study, this effect was not very clear for the compressive strength of the zeolite-geopolymer hybrid materials.

**Figure 9:**
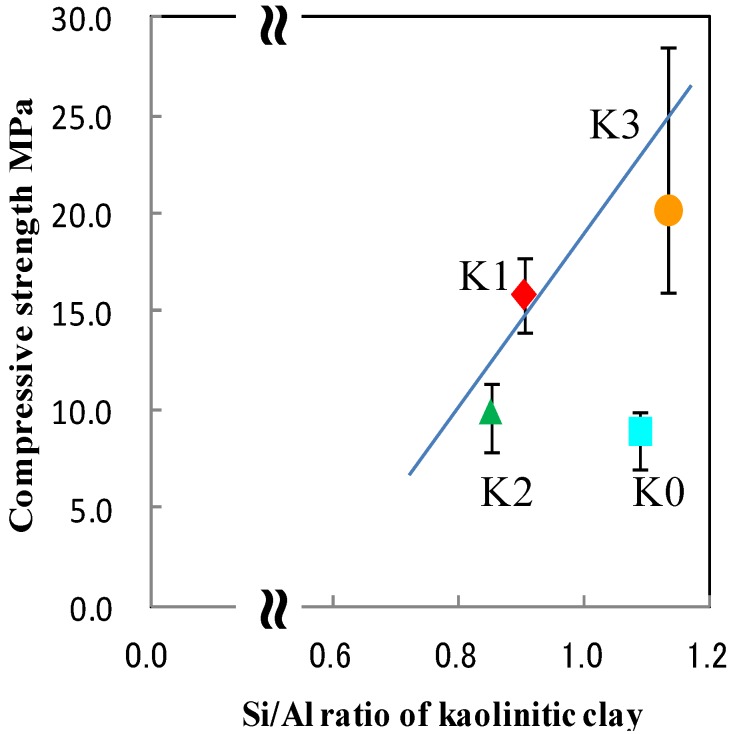
Relationship between the compressive strength and the Si/Al atomic ratio in the initial kaolinitic clays.

Figure 9 shows the variation in compressive strength with Si/Al atomic ratio in the initial kaolinitic clays. The compressive strength of the final bulk materials increased with increasing Si/Al ratio, in agreement with the results of Lizcano *et al.* [16,17,18]. BK0 did not fit this trend, likely because of the difference in crystal structure between halloysite and kaolinite.

**Figure 10:**
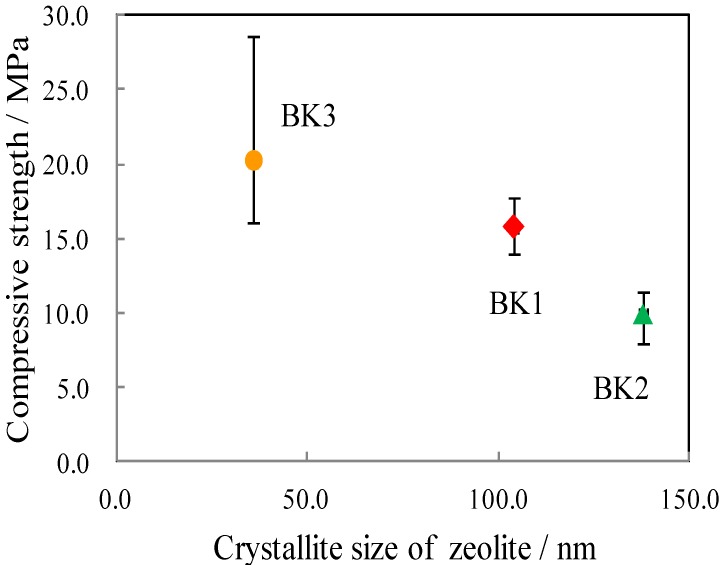
Relationship between the compressive strength and the crystallite size of zeolite in the final bulk materials.

Figure 10 shows the relationship between the compressive strength and the crystallite size of the synthesized zeolite. The compressive strength of the bulk materials decreased with increasing zeolite crystallite size, which suggests that micromorphological features is also an important factor for the compressive strength of the product. The coexistence of other crystal phases such as quartz, cristobalite, sericite, and other impurities in the starting kaolinitic clays could affect the compressive strength of the bulk materials. The role of the other crystal phases should be further investigated.

**Figure 11:**
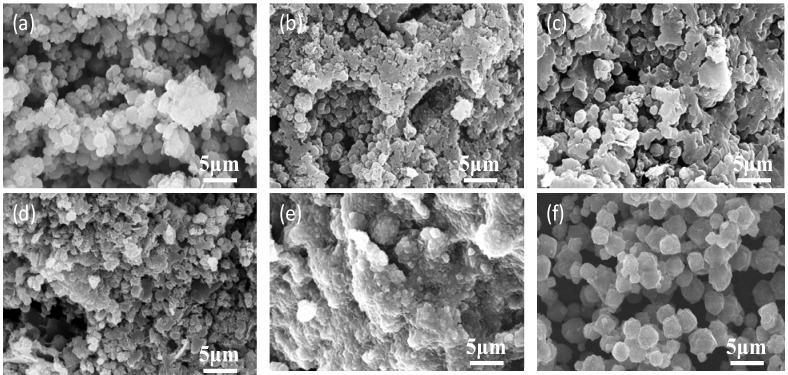
SEM micrographs of final bulk bodies (**a**) BK0; (**b**) BK1; (**c**) BK2; (**d**) BK3; (**e**) BK3Z; and (**f**) commercial X-type zeolite.

Figure 11 shows the microstructure of BK0, BK1, BK2, BK3, BK3Z, and commercial X-type zeolite. Particles of 1–2 μm in diameter were observed in BK0, BK1, BK2, and BK3. On the basis of (f) the morphology of commercial X-type zeolite; and (a) the formation of above 80% P-type zeolite, these particles were considered to be zeolite. In (e) BK3Z, these particles were less observable than in (d) BK3, and (f) the added commercial X-type zeolite was not observed. Thus, the added commercial X-type zeolites disappeared and new zeolite formed during the alkali-activation treatment. According to Figure 10, the compressive strength of the bulk materials decreased with increasing crystallite size of zeolite. As the zeolite crystals increased in size, it became easier to distinguish them as in the case of BK2 shown in Figure 11c. In contrast, smaller zeolite crystals coalesced, as in BK1 and BK3 (Figure 11(b,d) respectively), resulting in an increase in compressive strength.

**Figure 12:**
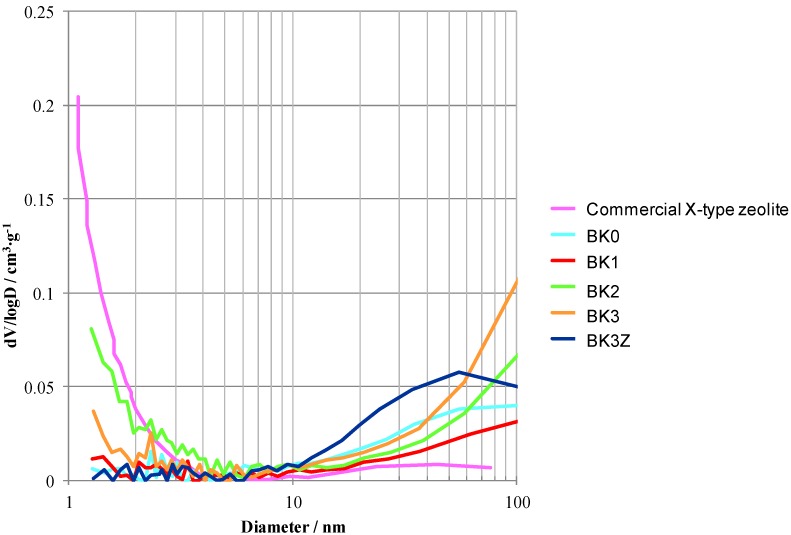
Pore size distribution of commercial X-type zeolite and final bulk bodies (BK0, BK1, BK2, BK3, and BK3Z).

Figure 12 shows the pore distribution of BK0, BK1, BK2, BK3, and BK3Z. BK3Z did not have any micropores smaller than 2 nm. The commercial X- type zeolite contained some mesopores smaller than 3 nm. The BK3Z results suggest that most of the commercial X-type zeolite, added into the starting slurry, decomposed because of the strong alkali solution used for the geopolimerisation. In contrast, BK2 had some nanoporosity due to the newly formed X-type zeolite. This X-type zeolite can absorb organic gas molecules or ionic elements in solution. Especially in BK2, many mesopores larger than 3 nm were detected, which may explain relatively lower compressive strength of the product. Tomura* et al.* reported that 3.2–7.4 nm pores can maintain the relative pressure of humidity between 40% and 70% which is the preferable range for the living environment for humans [19]. Therefore, BK2 is expected to be used as a humidity control material.

## 4. Conclusions

The relationship between the properties of initial kaolinitic clays, their metakaolin, and the final zeolite-geopolymer hybrid bulk material products was investigated. The specific surface area of metakaolin increased the amount of zeolite in the final products. The amount of zeolite did not show a clear correlation with the compressive strength of the zeolite-geopolymer hybrid bulk materials as previously reported. The atomic ratio of Si/Al in the kaolinitic clays and the crystallite size of the X-type zeolites produced indicated a clear correlation with the compressive strength of the hybrid bulk materials.
